# Socio-demographic factors associated with medication adherence among People Living with HIV in the Kumasi Metropolis, Ghana

**DOI:** 10.1186/s12981-022-00474-z

**Published:** 2022-11-14

**Authors:** Collins Adu, Kofi Akohene Mensah, Bright Opoku Ahinkorah, Dorothy Osei, Andrews William Tetteh, Abdul-Aziz Seidu

**Affiliations:** 1 Department of Health Promotion, Education and Disability Studies, Kwame Nkrumah University of Science and Technology, Kumasi, Ghana; 2 Department of Health Policy, Management and Economic, Kwame Nkrumah University of Science and Technology, Kumasi, Ghana; 3 School of Public Health, Faculty of Health, University of Technology Sydney, Sydney, Australia; 4 College of Public Health, Medical and Veterinary Sciences, James Cook University, 4811 Townsville, QLD Australia; 5 Centre For Gender and Advocacy, Takoradi Technical University, P.O.Box 256, Takoradi, Ghana

**Keywords:** Antiretroviral therapy, Human Immunodeficiency Virus/Acquired Immune Deficiency Syndrome, Medication adherence, Kumasi metropolis, Ghana

## Abstract

**Background:**

Medication adherence is important to the survival of People Living with HIV (PLHIV) globally. Although, HIV viral load is reduced by antiretroviral therapy (ART), the number of people on ART continues to rise in Ghana. In the Kumasi Metropolis, Ghana, we looked at the socio-demographic factors associated with medication adherence among PLHIV.

**Methods:**

A quantitative study involving 420 PLHIV who sought healthcare at the Kumasi South Regional Hospital was conducted utilizing a cross-sectional study design. We employed a structured questionnaire to collect data on medication adherence using the eight-item Morisky Medication Adherence Scale (MMAS) and socio-demographic factors that influence medication adherence. The data were analysed using Stata 14.2. Frequencies and percentages were used to present the descriptive data. The association between socio-demographic factors and medication adherence among PLHIV was investigated using both univariate and multivariate analyses.

**Results:**

More than half (53.10%) of PLHIV adhered to ART. Place of residence was significantly established to be influencing medication adherence among PLHIV. PLHIV who were residing in urban centers (aOR = 3.61; CI = 2.24–5.82) were more likely to adhere to medication as compared to those who resided in rural areas.

**Conclusion:**

Slightly more than half of PLHIV took their medicines as prescribed. Government and Policymakers such as the Ghana AIDS Commission, Ministry of Health, and Ghana Health Service should incorporate socio-demographic factors such as place of residence while creating and executing medication adherence initiatives to evaluate HIV management regimen for PLHIV.

## Background

Medication adherence plays a crucial role in the survival of People Living with HIV (PLHIV) globally as HIV/AIDS continues to be an issue of public health importance. HIV, which was discovered in 1981, has claimed a lot of lives to date [1]. In 2013, it was estimated that HIV affected about 78 million people with about 39 million dying of the disease since it was discovered [2]. Only 16.3 million of Africa’s 25.7 million PLHIV receive antiretroviral therapy (ART) services [3]. Additionally, in 2018, there were 470,000 deaths from HIV/AIDS in Africa [3]. Sub-Saharan Africa (SSA) is home to around 52% of PLHIV [4]. There is a higher prevalence of HIV within the sub-Saharan African region than the global average of 0.8% [5]. The fatality rate from HIV/AIDS is still high, and a sizable minority of PLHIV are still not receiving ART [6]. Only 39% of the 2.6 million ART-treated PLHIV in Western and Central Africa had suppressed viral levels, which suggests non-adherence [7]. In Ghana, ever since HIV was identified in 1986 [4], there has been a considerable decline in its prevalence. The number of persons on ART continues to rise [8]. At the end of 2018, there were an estimated 334, 713 (1.69%) PLHIV living in Ghana [9]. Of them, 19.9% of children under the age of 15 and 35.16% of adults over the age of 15 were receiving ART [9]. It was also found that 14,000 persons died in Ghana from AIDS-related causes of which 11,000 were adults over the age of 15 [10]. HIV prevalence in Ghana was 2.0% in 2014, down from 2.2% to 2006, according to the Ghana Demographic and Health Survey (GDHS) [11].

Since, medication adherence has been the focus of many researchers, HIV/AIDS is evolving into a chronic illness that can be managed. The National AIDS Control Programme (NACP) of Ghana has introduced maximization of adherence to medication as one of the strategies in the fight against the disease [12]. The definition of the word ‘adherence’ is very complex, encompassing adherence to nutritional requirements, scheduled visits (time), and dosage (medication) [13]. Medication adherence is described as taking all medications as prescribed by a healthcare provider at the correct time and in the correct amount [14, 15]. The introduction of ART has reduced HIV related rates of mortality and morbidity internationally [16]. It is always recommended that ART is introduced immediately after a person is diagnosed with the disease [17]. Evidence suggests that ART helps to delay the progression of HIV infection to AIDS and therefore slowing the disease’s spread across communities [18]. Medication adherence is critical for optimal management [19]. Bezabhe et al. [20] found that PLHIV on ART should be instructed to achieve a medication adherence rate of at least 95%. In addition to the maximal viral suppression when medication adherence is ≥ 95% [19], the number of opportunistic infections decrease [21]. Noncompliance with medication is linked to the development of drug-resistant HIV strains, recurrent hospitalization, and, eventually, mortality. [17, 21, 22].

Several studies conducted in Asia [23], SSA [23, 24] and in specific countries, including China [25], Botswana [26], and Nigeria [27] have indicated that there have been variations in medication adherence among PLHIV. This suggests that there are various national and regional disparities as far as adherence is concerned. Therefore, the findings of those studies conducted may not be applicable in the context of Ashanti Region, Ghana. Previous studies on adherence to medication in Ghana indicated adherence to medication rate at 62.2% in 2013 [28], 51.4% in 2014 [29], 67% in 2015 [5], 75% in 2017 [30], and 73% in 2018 [31]. These studies were conducted in Western Region, Central Region, Upper West Region, Volta Region, and Eastern Region of Ghana respectively. However, due to cultural differences that may differ between geographical zones of the country, these statistics conceal many geographical discrepancies. Evidence from the previous studies reviewed indicates that none of such studies has been conducted in the Kumasi Metropolis in the Ashanti Region of Ghana. In light of this, the purpose of this study was to determine the socio-demographic factors that influence medication adherence among PLHIV seeking healthcare at the Kumasi South Regional Hospital. The outcome of the study will be of great importance to the hospital, the region, and the country at large. The study’s findings will help close the knowledge gap, aid the Ministry of Health and other key players in developing policies, and evaluate HIV management recommendations.

## Methods

### Study design and setting

This study employed a quantitative method using a descriptive cross-sectional study design [32]. Data were collected from the month of October to December 2020, using a structured questionnaire. To aid in the data collection, the lead investigator trained three research assistants. To further maintain confidentiality, the data collection took place in a private area at the chosen ART centre. The questions on medication adherence were asked in the local dialect (Twi) which was then translated into English. It took 15 to 30 minutes to finish the questionnaire on each respondent. All of the questionnaires were checked for accuracy and completeness at the conclusion of each data collection session. Up until the study’s required sample size was reached, this process was continued. Prior to the commencement of the study, pre-testing of the data collection instrument was carried out among 20 PLHIV at Suntreso Government Hospital to verify the data collection tool’s authenticity and reliability.

The research was carried out at the Kumasi South Hospital, a regional hospital in the Ashanti Region of Ghana. This regional hospital is one of the few in the region that offers ART treatment to PLHIV in Ghana. The hospital is at the center of the region, it is raised 250 and 300 m above sea level, and it is settled between longitude 1.30^0^ West and latitude 6.35^0^ North and 6.40^0^ South [11]. Kumasi South Regional Hospital was constructed in 1976, as changed to be the Kumasi South later changed to be the Kumasi South Hospital. In 2002, the hospital was uprated to the status of a regional hospital. The regional hospital, a national health insurance scheme accredited facility, has a 140-bed capacity. Kumasi South Regional Hospital is located in between Agogo and Chirapatre, within the Kumasi Metropolis. About 30.3% of the population in Kumasi Metropolis is in Asokwa where Kumasi South Hospital is located [11].

Kumasi South Hospital is surrounded by seventeen health facilities. Among these facilities, CHAG and one government owned facility each and the remaining are privately owned. In 2017, HIV/AIDS-related conditions were recorded among top ten OPD morbidities. The hospital provides STI, VCT, and ART services for PLHIV.

### Sample size

A sample of 420 PLHIV receiving ART was selected using Snedecor and Cochran’s formulae [33] by taking a sample of 0.5% of PLHIV receiving ART and adhering to their medication from a study conducted in Ghana’s Upper West Regional Hospital [28], a 95% confidence level, a 5% margin of error and a 10% non-response rate.

The sample size of the study population was calculated using the Snedecor & Cochran’s formulae [33]:

$$n=\frac{{z}^{2}pd}{{d}^{2}}$$,

where n is the sample size.

$$z = standard deviation$$

, fixed value at 1.96 for 95% confidence interval.

p = proportion of PLHIV on adherence to medication 0.5 (According to research done at the Upper West Regional Hospital in Ghana, the projected proportion of PLHIV who adhered to ART (62.2%) [28].

$$q=1 - p$$

d = degree of accuracy desired, 0.05.

$$n =\frac{{\left(1.96\right)}^{2 }\times 0.5 \left(0.5\right)}{{0.05}^{2}}$$

$$n = \frac{0.964}{0.0025}$$

$$n = 382$$

An additional 10% of sample size was added for non-response.

$$\frac{10}{100}\times 382$$

$$n=38+382$$

$$n=420$$

Therefore, the sample size for this study was set at 420.

### Population, eligibility criteria and sampling

The study participants were PLHIV seeking treatment at the Kumasi South Hospital in Ghana. At the time of the study, there were 4,235 PLHIV seeking medical attention at the Kumasi South Hospital. PLHIV, above the age of 18, had used ART for at least six months, and gave their agreement to participate were considered eligible for this study. PLHIV who were seriously ill or admitted during the data collection were not included in the study. A sample size of 420 PLHIV and were on ART completed the survey that gave a response rate of 100% (420/420). The respondents were sampled using a basic random sampling method based on the lottery method. Numbers 0 and 1 were written on each piece of paper totaling 840 and folded into a polythene bag using the lottery method. During the data collection period, eligible participants were allowed to pick one of the folded papers in the polythene bag at random based on their average daily attendance at the clinic. Eligible participants who picked a sheet of paper with 1 written on the sheet were selected to take part in the study. This act was repeated continuously until the required sample size was attained.

### Definition of variables

#### Outcome variable

Medication adherence among PLHIV was the study’s outcome variable. The eight item Morisky Medication Adherence Scale (MMAS-8) questionnaire was used to classify high and low medication adherence. The MMAS-8 is a self-report medication adherence scale with eight items. The scale has good psychometric qualities, according to previous studies [15, 34]. The MMAS-8 was created to provide information on medication-related behaviors that are both unintentional and intentional [35]. The study incorporated the classification of medication adherence into high and low levels of medication adherence from a prior study carried out in India [36].

#### Explanatory variables

In our estimations, we used nine explanatory variables for the study. These variables included age, gender, marital status, ethnicity, work status, education level, place of residence, religious affiliation, and monthly income. None of these variables were chosen at random; rather, they were chosen based on the findings of previous studies on medication adherence, as well as their theoretical and conceptual implications for HIV/AIDS [25, 31].

### Statistical analyses

We employed both descriptive and inferential analytical approaches. During data collection, questionnaires were reviewed for completeness and internal problems. Questionnaires were numbered, sorted, and kept in files. Stata version 14.2 was used to analyze the data. The analysis was done in two main steps. The first step was the description of the sample and prevalence of medication adherence using frequencies and percentages. Second, the factors associated with medication adherencewere assessed using a binary logistic regression analysis (see Table 2) and the results were presented as crude and adjusted odds ratios(aOR) with their respective 95% confidence intervals indicating precision. For all tests, the statistical significance level was set to p <0.05.

**Table 1:** Socio-demographic information of PLHIV

Variable	Categories	Frequency(n = 420)	Percent (%)
**Age**	18–23	11	2.62%
	24–29	26	6.19%
	30–35	47	11.19%
	36–41	81	19.29%
	> 41	255	60.71%
**Gender**	Male	84	20.00%
	Female	336	80.00%
**Marital status**	Married	176	41.90%
	Single	117	27.86%
	Divorced	54	12.86%
	Widowed	73	17.38%
**Ethnicity**	Akan	304	72.38%
	Ewe	23	5.48%
	Ga	7	1.67%
	North	86	20.48%
**Employment status**	Yes	341	81.19%
	No	79	18.81%
**Level of education**	None	85	20.24%
	Primary	45	10.71%
	JHS/Middle	263	62.62%
	Tertiary	27	6.43%
**Place of residence**	Urban	285	67.86%
	Rural	135	32.14%
**Religious affiliation**	Christianity	369	87.86%
	Islamic	47	11.19%
	Traditional	4	0.95%
**Income (**GH$\not\subset$)	< 500	79	18.81%
	500–1000	292	69.52%
	> 1000	49	11.67%

### Ethical approval/consideration

The Committee on Human Research, Publications, and Ethics at Kwame Nkrumah University of Science and Technology was first consulted for ethical approval (approved protocol number CHRPE/AP/363/20, approval date October 8, 2020). Permission was also requested from the regional hospital’s administration. After a verbal and written explanation of the methods and risks involved using an information sheet, all participants and subjects were given a consent form. Those who agreed to be part of this study were made either to sign or thumbprint a consent form.

## Results

**Fig. 1:**
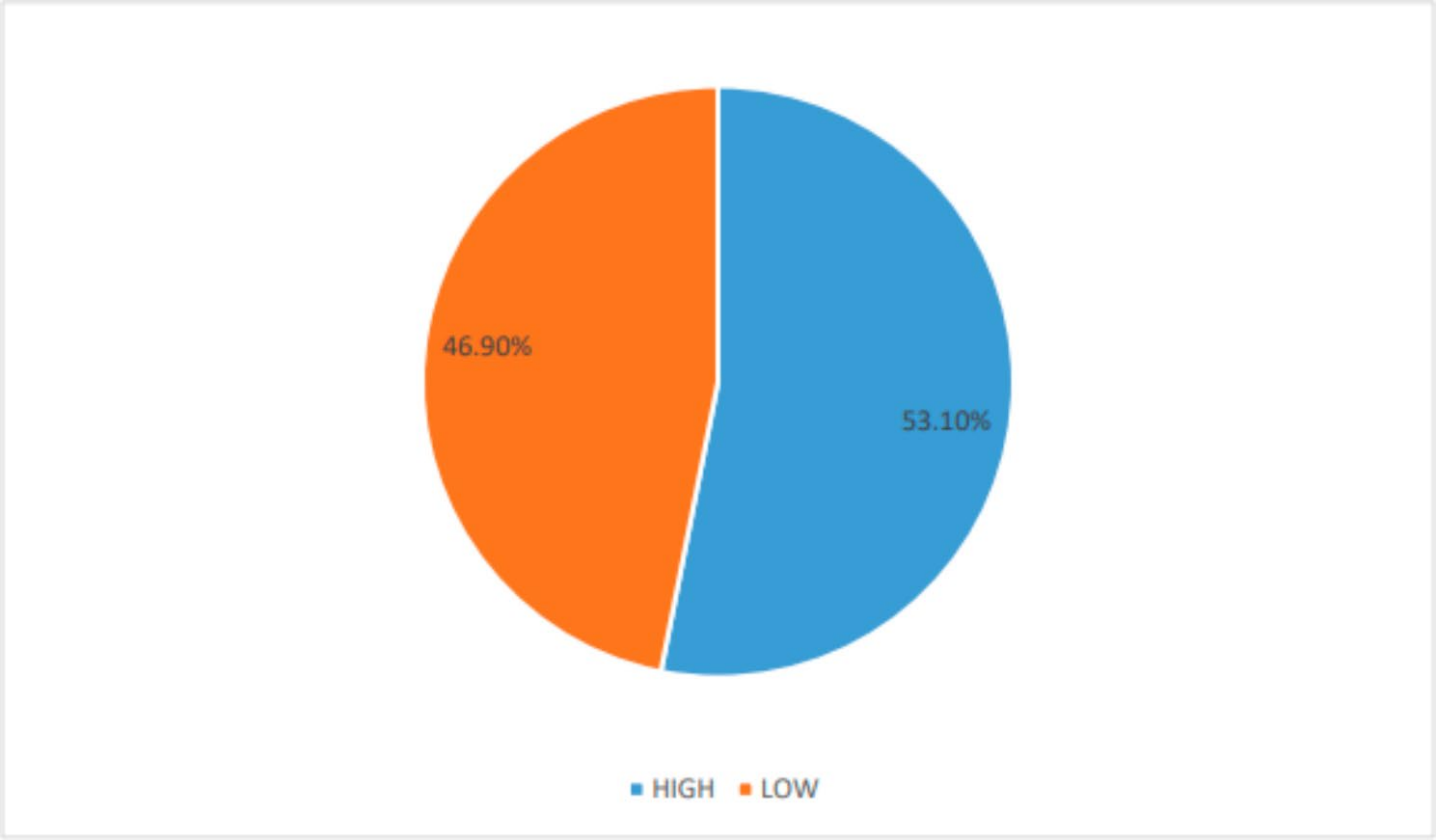
Medication adherence among PLHIV

Figure 1 shows the medication adherence among PLHIV in the Kumasi Metropolis in the Ashanti Region of Ghana. According to the MMAS-8, 223 (53.10%) of respondents accessing healthcare at Kumasi South Hospital in Ghana had high level of medication adherence and 197 (46.90%) had low medication adherence (See Fig. 1).

### Socio-demographic information of PLHIV in Kumasi Metropolis, Ghana

Table 1 displays the socio-demographic information of PLHIV. Majority of the PLHIV (80.00%) were females while the remaining (20.00%) were males. Also, most of the PLHIV (60.71%) were above the age of 41 with a mean age of 45 years ranging from 18 to 80 years. A higher proportion of PLHIV had JHS/Middle education (62.62%). Furthermore, the Akan (72.38%), PLHIV residing in urban centers (67.86%), Christians (87.86%), those who were working (81.19%), and those who had between GH$\not\subset$500 and GH$\not\subset$1000 as their income at the end of the month (69.52%) had the highest proportion of respondents (Table 1).

**Table 2:** Socio-demographic factors associated with medication adherence among PLHIV

Variables	Crude Odds Ratio	95% Confidence Interval			p-value	Adjusted Odds Ratio	95% Confidence Interval		p-value
**Gender**									
Male	Ref					Ref			
Female	0.81	0.50		1.30	0.38	1.09	0.62	1.92	0.77
**Age**									
18–23 24–29 30–35 36–41	Ref 0.78 0.99 1.20	0.18 0.25 0.33		3.43 3.88 4.44	0.74 0.99 0.78	Ref 0.42 0.49 0.65	0.86 0.11 0.16	2.05 2.91 2.73	0.28 0.35 0.56
> 41	1.97	0.56		6.89	0.29	1.40	0.35	5.56	0.63
**Marital status**									
Married	1.25	0.68		2.31	0.48	1.30	0.66	2.57	0.76
Single Divorced	1.04 Ref	0.54		1.98	0.92	1.33 Ref	0.64	2.78	0.45
Widowed	0.98	0.48		1.98	0.95	1.07	0.50	2.30	0.87
**Level of education**									
None	Ref					Ref			
Primary	0.68	0.33		1.41	0.30	0.83	0.37	1.82	0.64
JHS/Middle	0.78	0.48		1.28	0.33	0.77	0.48	1.34	0.36
Tertiary	1.00	0.42		2.39	0.99	1.06	0.40	2.82	0.91
**Religion**									
Christianity	2.68	0.28		25.97	0.40	2.01	0.15	26.38	0.59
Islamic	2.64	0.26		27.26	0.42	1.10	0.12	23.27	0.71
Traditional	Ref					Ref			
**Place of residence**									
Urban	3.22	2.07		5.01	**< 0.00**1	3.61	2.24	5.82	**< 0.001**
Rural	Ref					Ref			
**Tribe**									
Akan	1.23	0.27		5.60	0.79	0.54	0.28	8.47	0.62
Ewe	0.86	0.15		4.76	0.86	0.94	0.14	6.34	0.95
Ga	Ref					Ref			
North	1.16	0.23		5.24	0.90	1.69	0.28	9.99	0.57
**Employment status**									
Yes	1.29	0.79		2.12	0.31	1.23	0.69	2.20	0.48
No	Ref					Ref			
**Income (**GH$\not\subset$)									
< 500	1.00	0.49		2.05	0.99	1.32	0.61	2.85	0.48
500–1000	0.70	0.38		1.28	0.25	0.91	0.46	1.80	0.79
> 1000	Ref					Ref			

### Socio-demographic factors associated with medication adherence among PLHIV in Kumasi Metropolis, Ghana

Table 2 presents the socio-demographic factors associated with medication adherence among PLHIV in Ghana. With the univariate and multivariate regression analysis, the results showed that place of residence was significantly associated with medication adherence among PLHIV as detailed in Table 2. For instance, respondents who were residing in urban centers (aOR = 3.61; CI = 2.24–5.82) were more likely to adhere to their medication compared with those who were residing in rural areas.

## Discussion

In this study, we looked at the relationship between socio-demographic factors and medication adherence among PLHIV in the Kumasi Metropolis, Ghana. Medication adherence was observed to be 53.10% among PLHIV. This study’s level of medication adherence is higher than previous study conducted in the Volta Region of Ghana [29], which reported 51.4% rate of medication adherence among PLHIV. Also, previous studies conducted in Central Region of Ghana [31], Eastern region of Ghana [30], Upper West region of Ghana [28], and Western region of Ghana [5] recorded higher rates of medication adherence to be 73%, 75%, 62.2%, and 67% respectively. However, the World Health Organization (WHO) and national requirements of medication adherence of 95% or above are not met by the study’s reported medication adherence rate [9, 37]. Medication adherence rates in SSA are quite inconstant and often pathetic, and a large number of patients are likely to progress to the AIDS stage when adherence is suboptimal [27].

Understanding the socio-demographic factors that influence medication adherence among PLHIV could aid in the successful implementation of adherence intervention programs [31]. The study found that where people lived had a substantial impact on their adherence to their medications. In this study, PLHIV in urban areas were found to be more likely to adhere to their medication. Prior studies in Ethiopia [38] and Kenya [39] support this finding. This study, on the other hand, contradicts prior study conducted in SSA, which found that living in a city encourages non-adherence to medication [40]. One possible explanation is that PLHIV in rural locations have a poor socioeconomic position [38]. Similarly, PLHIV who live in rural areas have a low income. This could lead to poor medication adherence. PLHIV in urban areas use ART services on a frequent basis due to their closeness to a health institution. The bulk of health facilities in Ghana that offer ART services are in urban locations, and PLHIV are expected to go to these places for treatment. As a result, PLHIV from rural areas may miss appointments and have poor medication adherence.

### Strengths and limitations

It is critical to explain the current study’s conclusions in terms of the study’s strengths and limitations. Standard data collection methods, including the utilization of experienced researchers, were used in the survey’s methodology and data collection process, resulting in a high response rate. Furthermore, we used advanced statistical tools to analyze the data. Also, we employed a standardied instrument (MMAS) in assessing medication adherence. This ensured that the data were thoroughly examined. Despite these strengths, the study also had some limitations. We cannot establish causal correlations between the factors investigated because of the cross-sectional study design. Secondly, the real reflection of respondents’ adherence to ART and any potential barriers may not have been captured since the study employed quantitative study design and did not give room for respondents to explain their reasons. The sensitive nature of the investigation may have made social desirability bias more prevalent.

## Conclusion

According to the findings, about 53 out of every 100 PLHIV adhered to their medication. Place of residence was significantly associated with medication adherence among PLHIV. When designing and implementing programs on medication adherence among PLHIV in Ghana, policymakers such as the Ghana AIDS Commission, Ministry of Health, Ghana Health Service, and National AIDS Control Programme should include socio-demographic factors while creating and executing medication adherence initiatives to evaluate HIV management regimen for PLHIV. Qualitative research is required to acquire a better understanding of how the highlighted factors affect the medication adherence of PLHIV.

## Data Availability

The datasets used and/or analyzed in the current study are available from the corresponding author on reasonable request.

## List of abbreviations

SSA: Sub-Saharan Africa
VCT: Voluntary Counselling and Testing
ART: Antiretroviral Therapy
aOR: adjusted Odds Ratio
HIV: Human Immunodeficiency Virus
AIDS: Acquired Immunodeficiency Syndrome
CHAG: Christian Health Association of Ghana
OPD: Outpatient Department
STI: Sexually Transmitted Infection
NACP: National AIDS Control Programme
MMAS-8: Morisky Medication Adherence Scale
GH$\not\subset$: Ghana Cedi
PLHIV: People Living with HIV
WHO: World Health Organization
